# Diffused Intra-Oocyte Hydrogen Peroxide Activates Myeloperoxidase and Deteriorates Oocyte Quality

**DOI:** 10.1371/journal.pone.0132388

**Published:** 2015-07-21

**Authors:** Sana N. Khan, Faten Shaeib, Tohid Najafi, Mahendra Kavdia, Bernard Gonik, Ghassan M. Saed, Pravin T. Goud, Husam M. Abu-Soud

**Affiliations:** 1 Department of Obstetrics and Gynecology, The C, S, Mott Center for Human Growth and Development, Wayne State University School of Medicine, Detroit Michigan, United States of America; 2 Department of Biochemistry and Molecular Biology, Wayne State University School of Medicine, Detroit, Michigan, United States of America; 3 Department of Biomedical Engineering, Wayne State University, Detroit, Michigan, United States of America; 4 Department of Obstetrics and gynecology, Division of Reproductive Endocrinology and Infertility, University of California Davis, Sacramento, California, United States of America, and California IVF Fertility Center, Davis and Sacramento, California, United States of America; China Agricultural University, CHINA

## Abstract

Hydrogen peroxide (H_2_O_2_) is a relatively long-lived signaling molecule that plays an essential role in oocyte maturation, implantation, as well as early embryonic development. Exposure to relatively high levels of H_2_O_2_ functions efficiently to accelerate oocyte aging and deteriorate oocyte quality. However, little precise information exists regarding intra-oocyte H_2_O_2_ concentrations, and its diffusion to the oocyte milieu. In this work, we utilized an L-shaped amperometric integrated H_2_O_2_-selective probe to directly and quantitatively measure the real-time intra-oocyte H_2_O_2_ concentration. This investigation provides an exact measurement of H_2_O_2_ in situ by reducing the possible loss of H_2_O_2_ caused by diffusion or reactivity with other biological systems. This experiment suggests that the intra-oocyte H_2_O_2_ levels of oocytes obtained from young animals are reasonably high and remained constant during the procedure measurements. However, the intra-oocyte H_2_O_2_ concentration dropped significantly (40-50% reduction) in response to catalase pre-incubation, suggesting that the measurements are truly H_2_O_2_ based. To further confirm the extracellular diffusion of H_2_O_2_, oocytes were incubated with myeloperoxidase (MPO), and the diffused H_2_O_2_ triggered MPO chlorinating activity. Our results show that the generated hypochlorous acid (HOCl) facilitated the deterioration in oocyte quality, a process that could be prevented by pre-incubating the oocytes with melatonin, which was experimentally proven to be oxidized utilizing HPLC methods. This study is the first to demonstrate direct quantitative measurement of intracellular H_2_O_2_, and its extracellular diffusion and activation of MPO as well as its impact on oocyte quality. These results may help in designing more accurate treatment plans in assisted reproduction under inflammatory conditions.

## Introduction

Hydrogen peroxide (H_2_O_2_) is a signaling molecule that comprises an important component of the oocyte microenvironment, and its overproduction mediates oocyte deterioration and dysfunction [1, 2] Hydrogen peroxide is typically generated from a nonenzymatic substrate decay or superoxide dismutase-catalyzed reaction of superoxide (O_2_^•−^) [3]. The biological effects of H_2_O_2_ are governed in part, by its intrinsic stability in the intracellular space, reactivity, oxidizing and reducing properties, permeability through cell membrane, function as a precursor of the more toxic hydroxyl radical, and a substrate for multiple enzymes [3, 4]. We and others have previously shown the critical role of this molecule in the female reproductive system, in that exogenously added H_2_O_2_ accelerates the aging process and deteriorates oocyte quality in concentration and time dependent manners [1, 5, 6]. These findings suggest that direct and precise intracellular measurements of H_2_O_2_ and its diffusion through the oocyte membrane are critically important, specifically under certain inflammatory conditions.

Given the importance of physiologic concentrations of H_2_O_2_ in signaling and defense as well as the potential for damage at elevated concentrations, the balance of the production and removal, either by scavenging or transportation out of the cell is paramount to the maintenance of the overall biological redox status [7]. Previously it was widely believed that H_2_O_2_ was able to freely cross biological membranes, this was likely to explain observed phenomenon as opposed to experimental data [7]. Recently; however, newer data suggests that membranes have limited permeability to H_2_O_2_, which suggests that the molecular transportation must occur via means other than unhindered diffusion [8, 9]. The permeability of a membrane to H_2_O_2_ could be altered by changes in membrane composition, stage of cell cycle or by mechanical forces exerted on biological membranes [7]. Although numerous studies have focused on the exogenous addition of H_2_O_2_ in the deterioration of oocyte quality [1, 5, 6] information focused on its intracellular production or its transportation in the oocyte microenvironment and how these factors affect oocyte quality are lacking.

Since myeloperoxidase (MPO), an inflammatory extracellular enzyme, utilizes H_2_O_2_ as a substrate, the ability of H_2_O_2_ to diffuse to the extracellular environment is of critical importance when inflammatory changes disrupt the normal milieu [10–12]. Myeloperoxidase is a homodimeric heme-containing enzyme found in azurophilic granules of neutrophils [13, 14]. In inflammatory conditions, MPO is released from the neutrophils in the extracellular environment where, in the presence of H_2_O_2_, it generates the toxic oxidant, hypochlorous acid (HOCl) through a catalytic cycle common to the mammalian peroxidase family. Derangements in MPO have also been implicated in many inflammatory conditions such as diabetes and cardiovascular disease as well as reproductive disorders such as polycystic ovary syndrome, endometriosis, and ovarian cancer [10, 11, 15–19], although an understanding of the mechanism of these damaging effects is unclear.

Melatonin is known to play an important role in the homeostasis of various neuroendocrine systems including circadian sleep rhythms, blood pressure, immunity, and reproduction [20, 21]. Importantly, melatonin also has antioxidant functions as it is known to prevent lipid peroxidation and lipoprotein modification [21]. As a protective antioxidant, melatonin can prevent HOCl insult by directly scavenging HOCl or by modulating the catalytic activity of MPO by the catalytic oxidation of melatonin [10, 22, 23]. The capacity of melatonin to compete with Cl^-^ leads to the inhibition of the MPO chlorinating activity while maintaining the peroxidation activity of MPO [22–25]. We have recently shown that HOCl is able to accelerate oocyte aging and deteriorate oocyte quality to a higher degree than other oxidants such as O_2_^•−^ or H_2_O_2_ [1, 26].

The present studies evaluate whether intracellular H_2_O_2_ can serve as an extracellular substrate for MPO to produce HOCl thus deteriorating oocyte quality, and whether melatonin can protect against this damage. To accomplish these goals, an L-shaped amperometrically integrated H_2_O_2_-selective electrode has been modified to directly measure the H_2_O_2_ concentration continuously within non-untreated oocytes or oocytes treated with catalase. High performance liquid chromatography (HPLC) was used to understand the diffusion of H_2_O_2_, and its reaction with MPO to generate HOCl as well as the oxidation of melatonin. These findings have important implications for our understanding of the pathogenesis of inflammatory diseases and for the development of novel therapeutic strategies to combat inflammatory damage in the conditions with poor reproductive outcomes.

## Materials and Methods

### Materials

All the materials used were of the highest grade of purity and without further purification. Hydrogen peroxide, Human tubular fluid (HTF) media, melatonin (MEL), dimethylformamide (DMF), 3,3′,5,5′-tetramethylbenzidine (TMB), all the solvents used in HPLC experiment, anti-α tubulin antibody, FITC conjugate anti-goat antibody, propidium iodide, 1% BSA (Bovine Serum Albumin), 0.1% M Glycine, and 0.1% Triton X- 100 were obtained from Sigma–Aldrich (St. Louis, MO, USA). Normal Goat Serum (2%) was from Invitrogen (Grand Island, NY) and 0.2% Powder Milk from grocery. The study involved the use of oocytes obtained from super-ovulated 8–14 week-old mice B6D2F1 (n = 20), which was approved by Wayne State University's Animal Investigation Committee.

### Intra-oocyte H_2_O_2_ measurement

Cumulus oocytes retrieved from the oviductal ampullae were treated with 0.1% hyaluronidase (w/v) in Human tubular fluid (HTF) media (Sigma–Aldrich (St. Louis, MO, USA)) for 2–4 minutes at 37°C. Oocytes were subsequently denuded to remove all cumulus cells with a narrow bore pulled glass Pasteur pipette, thoroughly rinsed in M2 media (Sigma–Aldrich), then the oocytes screened for the presence of the polar body confirming their Metaphase II stage. Oocytes then kept in HTF medium (Sigma–Aldrich) pre-equilibrated with 5% CO_2_ in air at 37°C in a common pool before randomly transferred into test and control groups. Twenty non cumulus oocytes were used for H_2_O_2_ electrode experiment.

Non cumulus oocytes were pre-incubated with 100 μM melatonin in HTF media and treated with 40 nM MPO for 24 h. Of note, HTF media contains Cl^-^ levels akin to the oviductal fluid (~100mM). Treated oocytes were then subjected to indirect fluorescence immunocytochemistry to assess the alterations in metaphase-II mouse oocyte microtubules morphology (MT) and chromosomal alignment (CH) (markers of oocytes quality), and compared to untreated oocytes and oocytes incubated with melatonin (100 μM) alone for 24 h. In the same experiment, all the HTF media from the all treated and untreated groups was filtered and investigated using HPLC.

### Immunofluorescence staining and fluorescence microscopy

Immunofluorescence staining and fluorescence microscopy were performed as previously described [5, 27]. Images were obtained utilizing both immunofluorescence and confocal microscopy.

### Confocal microscopy, assessment of oocyte quality

Slides were examined with the Axiovert 25 inverted microscope (Zeiss, Thornwood, NY) using Texas Red (red) and FITC (green) fluorescent filters with excitation and emission wavelengths of 470 and 525 nm, and 596 and 613 nm, respectively. Confocal images were obtained utilizing a Zeiss LSM 510 META NLO (Zeiss, Germany) microscope as previously described [27]. Three independent observers blinded to the assigned treatment groups performed the categorization of oocytes based on MT and CH status. Observers used comprehensive evaluation of the individual optical sections and the 3-D reconstructed images.

### Myeloperoxidase Purification

Myeloperoxidase (MPO) was initially purified from detergent extracts of human leukocytes by sequential lectin affinity and gel-filtration chromatography [28–30]. Trace levels of contaminating eosinophil peroxidase were then removed by passage over a sulfopropyl Sephadex column [29]. Purity of isolated MPO was established by demonstrating a Reinheitzal value of 0.85 (A430/A280), SDS–PAGE analysis with Coomassie blue staining, and gel tetra- methylbenzidineperoxidase staining to the absence of contaminating eosinophil peroxidase activity. Enzyme concentration was determined spectrophotometrically utilizing extinction coefficients of 89,000 M^−1^ cm^−1^/heme of MPO [31].

### Melatonin solution

A stock solution of melatonin was dissolved in dimethylformamide (DMF) and then diluted to the required concentration with phosphate buffer (pH = 7.4). The final concentration of DMF in all melatonin solutions was fixed (less than 1%) and did not interfere with MPO activity nor did it have any effect on oocyte quality [26].

### Electrode Description

H_2_O_2_ measurements were performed utilizing an Apollo-4000 H_2_O_2_ meter (World Precision Instruments, Sarasota, FL) equipped with an L-shaped H_2_O_2_ electrode (ISO-HPO-100). The features of this electrode have been previously described [32]. The absolute H_2_O_2_-reactive part of the needle electrode compromises its proximal 5–15 μm with a diameter of 0.8–5 μm, which is insulated by glass and has 0.5 μM limit of detection of H_2_O_2_. The H_2_O_2_ sensor uses an Ag/AgCl reference electrode and the H_2_O_2_-selective membrane is a WPI (Worchester Polytechnic Institute—Worchester, MA USA) membrane and was not disclosed.

### Instrument Design

The electrode used in this study had a 10 μm H_2_O_2_ reactive part and a 2 μm diameter. The instrument calibration was performed at 37°C. The electrode was equilibrated and polarized along the recommendations of the manufacturing company (WPI). Special microtools were utilized for this procedure; these pipettes were made to meet the requirements of the measurements. For calibration, known amounts of H_2_O_2_ (0–2 μM) were used. The H_2_O_2_ (Sigma–Aldrich) solutions were prepared fresh in phosphate buffer (pH 7.4), then the concentration of the working solutions were determined spectrophotometrically (extinction coefficient of 43.6 M ^-1^ cm ^-1^ at 240 nm [5, 33, 34]. During the preparation process, all solutions were kept on ice to minimize decomposition. The calibration curve was constructed by plotting the signal output (pA) versus the concentration of the H_2_O_2_ (μM) added at that time (R^2^ = 0.997). The slope was then determined and entered into the Apollo 4000 software program to observe data in μM concentration mode.

A manipulated form of the aforementioned electrode was designed with a 45° angle curvature in the insulated part of electrode before insertion to increase solidification of the electrodes to prevent breakage of extremely fragile electrodes. By this method the response of the electrode is relatively fast due to its close proximity to the source of H_2_O_2_.

The H_2_O_2_-electrode was immersed into the phosphate buffered saline (PBS) buffer solution, the vial was placed over a plate stirrer, and the electrode allowed to stabilized for 3–5 min. Aliquots of different concentrations of H_2_O_2_ were added to the PBS buffer. The current (pA) output from the H_2_O_2_ electrode was increased rapidly. Within a few seconds the response reached a plateau and the second aliquot of H_2_O_2_ was then added. Successive additions of the remaining aliquots of H_2_O_2_ were made in a similar way.

### Procedure for in situ measurement

The entire procedure from oocyte preparation to H_2_O_2_ electrode insertion has been previously reported with the use of the oocyte media (human tubular fluid (HTF) media) or PBS buffer surface as the "zero point" [32]. The zona pellucida (ZP) was slit open using a partial zona dissection (PZD) micropipette, and the probe was inserted through the ZP opening deep into the ooplasm. The oolemma was broken after deep invigilation using a technique similar to Intra-cytoplasmic sperm injection (ICSI). The picoampere difference for the H_2_O_2_ signal and the corresponding micromolar difference in concentration were read off the mean of the H_2_O_2_ calibration curves. In addition to the control group of oocytes, an additional group (n = 20) was incubated with 100 nM catalase for 30–40 min during intraoocyte H_2_O_2_ measurement to ensure that the measurements were truly H_2_O_2_ based. To minimize errors, three factors were taken in consideration for catalase treatment: time of incubation, catalase concentration, and possible contamination with catalase. Reducing the catalase concentration to half with the same incubation time had little effect on the intra-oocyte H_2_O_2_ concentration. Therefore, the catalase concentration and incubation time were selected to assure maximum effect on the intra-oocyte H_2_O_2_ concentration. Additionally, dilution of the catalase solution to a lower concentration (< 5 nM) by adding fresh media prior to electrode insertion had no effect on the results indicating no contamination upon electrode insertion. Furthermore, in our experiments, no leaks occurred, and had clear membrane damage occurred, the oocyte would be excluded from the study.

### High-performance liquid chromatography analysis

HPLC analyses were performed using a Shimadzu HPLC system equipped with a SCL-10A controller, LC-10 AD binary solvent delivery pumps, SIL-10 AD autosampler, SPD-M10 A diode array detector and an RF-10 A XL fluorescence detector. The column used was an Alltech 5 μm particle size, 4.6 x 150 mm reverse phase octadecylsilica (C18). To monitor the chromatogram, the fluorescence detector was set at 321 nm for excitation and 465 nm for emission and the SPD diode array detector was set at 400 nm. HPLC grade solvents were prepared as follows: solvent A, 0.1% TFA in water and solvent B, 0.1% TFA (Trifluoroacetic acid) in 80% acetonitrile. Moreover solvent gradient was set as follows: 0–10 min 55–65% B, 10–14 min 65–90% B followed by reducing solvent B composition to 55% within 14–24 min. The column elution was carried out at a flow rate of 0.8 ml/min with the linear gradient of solvents. After incubation of melatonin (100 μM) with MPO (40 nM) in the medium containing mouse oocytes (n = 41) for 24 h, the reaction mixture was filtered through an Amicon Ultra-15 centrifugal filter unit with Ultracel-10 membrane (from Millipore) with a 3-kDa cut-off by centrifuging at 14,000 relative centrifugal force (rcf) for 30 min at 4°C [35]; then 50 μl of the filtered sample was injected. At the end of the run the system was equilibrated with 45% solvent A; each sample was analyzed in triplicate.

### Taurine Chloramine Assay

Eighty noncumulus oocytes were preincubated at 37°C in a buffer (containing 10 mM phosphate buffer, 10 mM potassium chloride and 140 mM sodium chloride, 1 mM calcium chloride, 0.5 mM magnesium chloride and 1 mg/ml glucose) with 5mM taurine [23] and 40 nM MPO. The control solution contained the same materials in the absence of oocytes. After 30 minutes, the solutions were centrifuged and the supernatants were put on ice. Formation of HOCl indicated MPO activity, and was assessed by a change in absorbance measurements using the taurine chloramine assay. Taurine Chloramine was assayed by adding 200 μl of the oocyte supernatant to 50 μl of a reagent solution containing 10 mM TMB and 100 μM sodium iodide in 50% DMF and 400 mM acetic acid [23]. Under these conditions taurine chloramine oxidizes TMB to a blue product with an absorbance maximum at 655 nm. A standard curve was performed by adding 50 μl of reagent to 200 μl of 10 mM phosphate buffer solution pH 7.4 containing taurine and different HOCl concentrations (0–50 μM). The absorbance measurements were detected in Spectra Max 190 plate reader (Molecular Devices).

### Statistical Analysis

Independent t-tests were performed using SPSS software version 22.0 (SPSS Inc., Chicago, IL, USA) to compare groups of oocytes treated with versus without catalase, statistical significance was indicated by P< 0.05.

## Results

**Fig 1:**
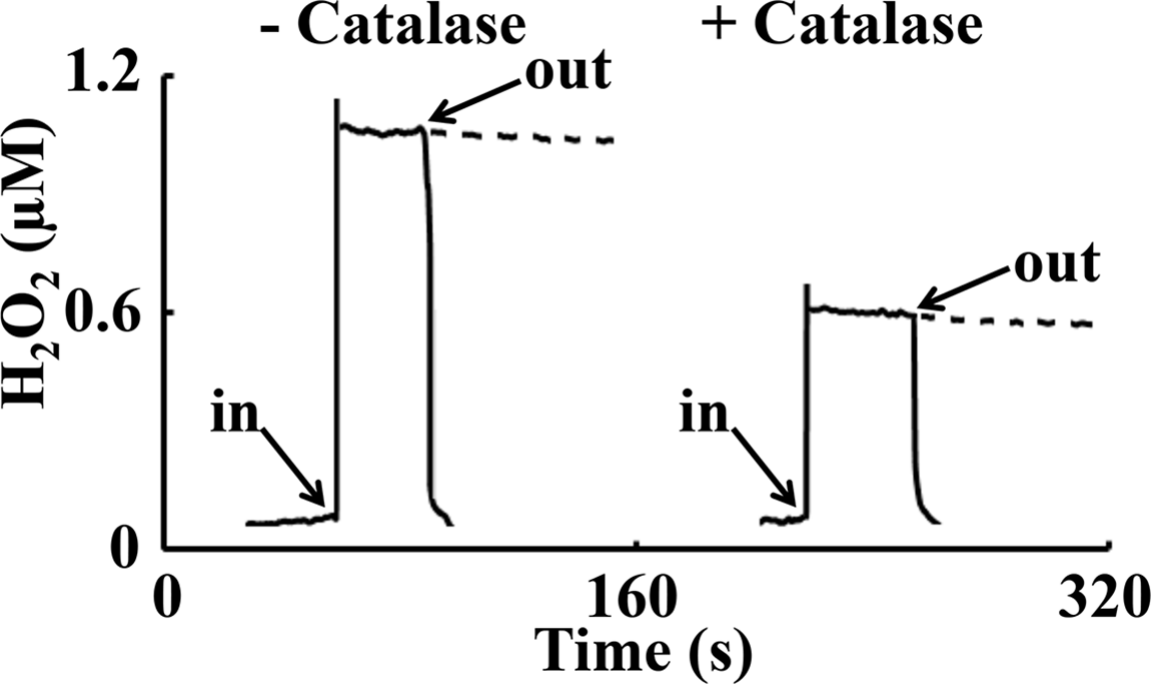
Real-time measurements of intra-oocyte H_2_O_2_ concentration utilizing H_2_O_2_-selective electrode. With the use of the oocyte media or PBS buffer surface as the “zero point”, the H_2_O_2_ electrode tip was inserted directly into the ooplasm. The pic o ampere differences were recorded and the stable intra-oocyte reading was taken as the H_2_O_2_ signal. The arrows show the time of insertion and withdrawal from the oocyte. The inset shows the insertion process.

**Fig 2:**
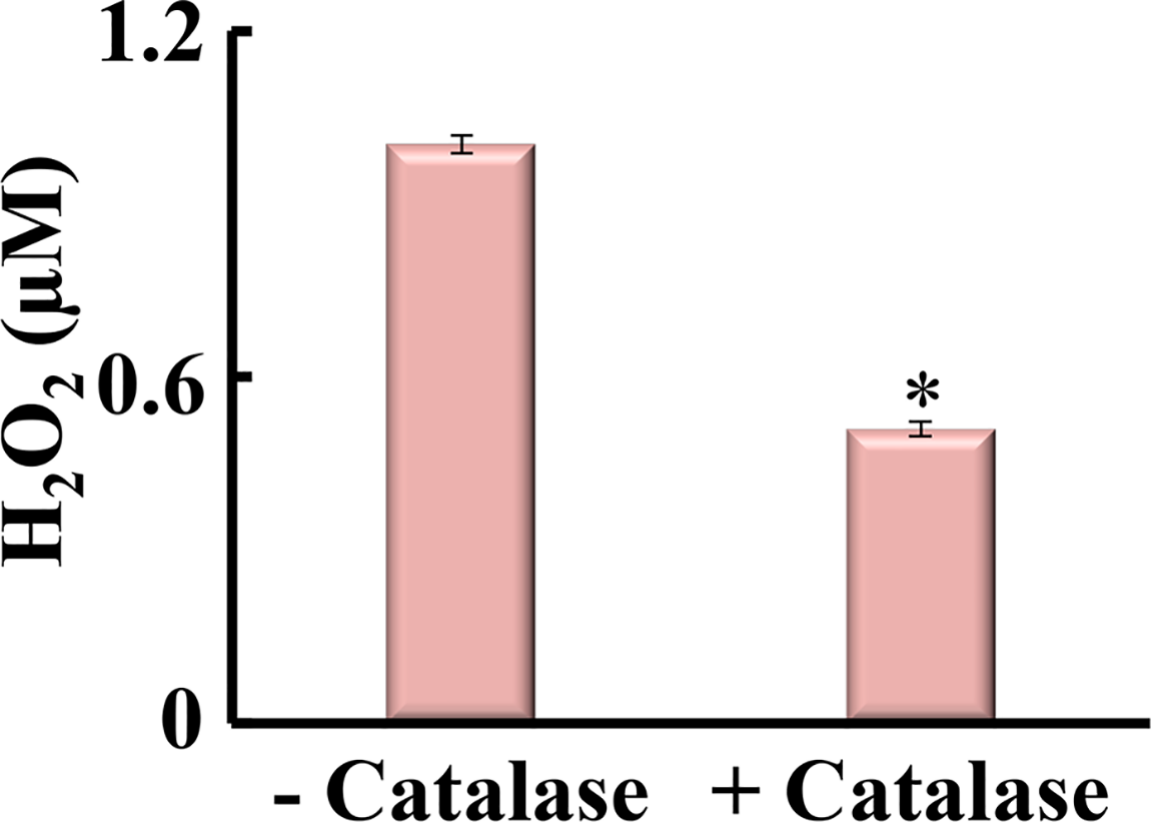
Effect of catalase, an H_2_O_2_ scavenger, on intra-oocyte H_2_O_2_ concentration (n = 20). The error bars represent the standard errors of mean.

An H_2_O_2_-sensitive electrode tip was inserted directly into untreated oocytes and oocytes treated with catalase, and the real-time profiling of intra-oocyte H_2_O_2_ *in vivo* were recorded. The arrows in Fig 1 show the time of insertion and withdrawal from two individual oocytes; incubated with 100 nM catalase for 30–40 min (Fig 1; right trace) versus untreated oocyte (left trace). As shown in Fig 2, in oocytes incubated with catalase (n = 20), the H_2_O_2_ fell significantly by ~50% compared with untreated oocytes (n = 20). These results confirm measurement of H_2_O_2_. The mentioned comparison was made using independent t-test for equality of means and Levene’s test for equality of variances. The mean intracellular H_2_O_2_ levels for control and catalase groups showed significant difference (P<0.001) (Fig 2).

The viability of oocytes was judged visually under 600× magnification using Nomarsky contrast during the measurement process. The parameters of oocyte quality [36–38] evaluated included: lack of intactness of their shape, darkness in cytoplasm membrane for oocytes undergoing lysis, and turgidity immediately after the electrode insertion after culture at 37°C under 5% CO_2_ in air for 1 hour. None of these signs were observed among the oocytes.

**Fig 3:**
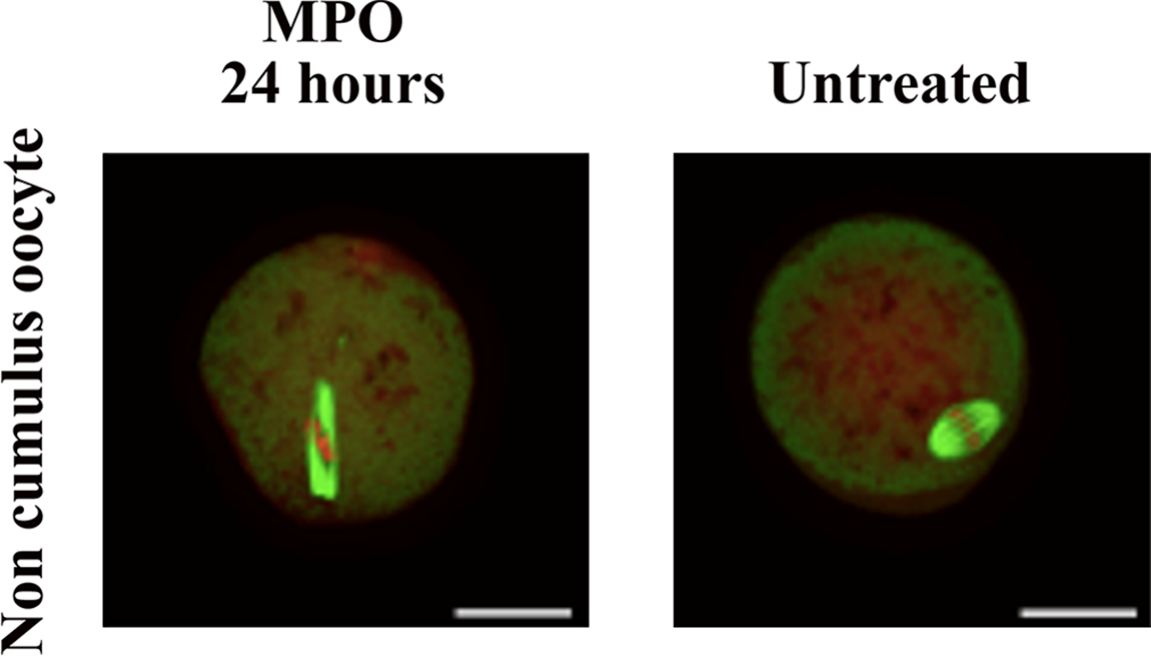
Effect of MPO on oocyte quality: The upper panel represents a noncumlus control oocyte with good quality scoring. The lower panel shows a noncumulus oocyte after incubation with MPO that received poor scoring.

Hypochlorous acid, the final product of MPO-Cl^-^-H_2_O_2_ system, is thought to be damaging to the oocyte, thus we thought to exploit the diffusion capacity of H_2_O_2_ and its ability to activate MPO that subsequently mediates deterioration of oocyte quality. The oocyte quality and viability was assessed visually before and after MPO/melatonin treatments. Our results showed poor outcomes for MT and CH in oocytes treated with MPO (40 nM) as compared to controls independent of presence of cumulus cells (Fig 3). No changes as compared with controls were visualized among the oocytes treated with a combination of MPO and melatonin for 3 hours (data not shown). Thus, a sufficient amount of H_2_O_2_ is diffused from the oocyte that is capable of activating MPO. Melatonin, a potent MPO inhibitor and HOCl scavenger, could attenuate these damages.

**Fig 4:**
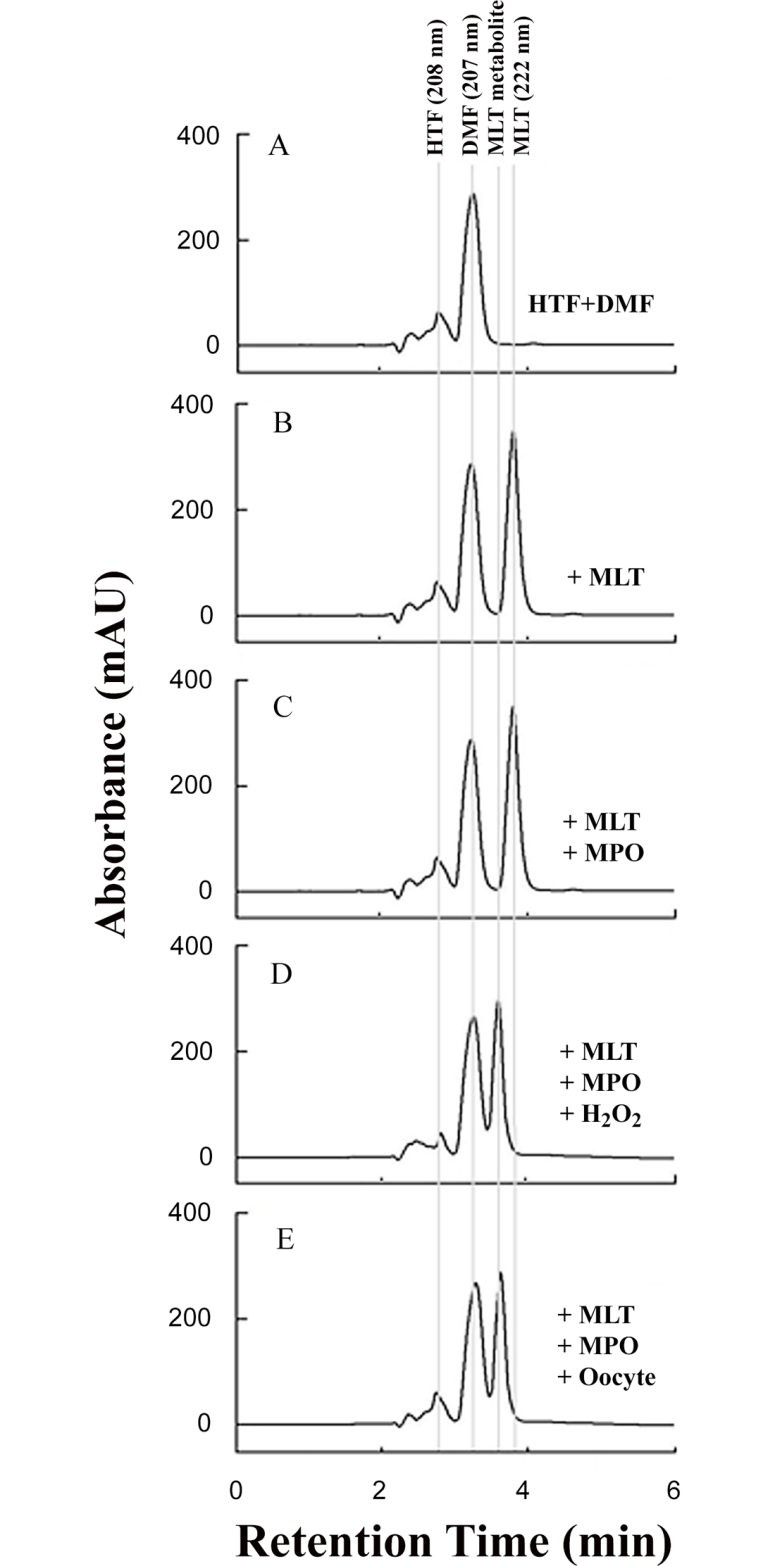
HPLC analysis shows evidence of the release of H_2_O_2_, which activates the catalytic function or MPO causing melatonin oxidation. A) HPLC trace DMF (elution time 3.31 min) and phosphate buffer (elution time 2.48 min). B) Trace for Melatonin (3.98 min) dissolved in DMF. C) Addition of MPO causes no significant change in melatonin peak intensity and/or retention time. D) Addition of exogenous H_2_O_2_ (sequential addition of 20 μM, total 200 μM) results in a significant shift in melatonin retention time elution time to a hydroxylated intermediate (3.71 min). E) Incubation of MPO and melatonin in the presence of oocytes produces similar peaks and retention time to exogenously added H_2_O_2_, signifying that H_2_O_2_ released from the oocytes reacts with MPO causing melatonin oxidation.

To investigate the mechanism of melatonin protection, HPLC analysis was performed under different experimental conditions; (1) melatonin alone (100 μM), (2) melatonin (100 μM) with MPO (40 nM), (3) melatonin (100 μM) with MPO (40 nM) treated with 100 μM H_2_O_2_, and (4) MPO (40 nM) with oocytes with melatonin (100 μM). Under our experimental conditions, melatonin (222 nm) eluted at 3.98 min and was identified by its characteristic spectra observed from the photodiode array detector (Fig 4A). MPO alone had no effect on melatonin elution time (Fig 4B). MPO incubated with melatonin in the presence of oocytes there was a progressive reduction in the melatonin signal along with the formation of new peak eluting at an earlier time (Fig 4C). Similar elution times were observed when melatonin was treated with MPO-Cl^-^-H_2_O_2_ system. Thus, our results suggest that, in the presence of oocytes, oxidation of melatonin is the result of the activation of the MPO system caused by the diffused H_2_O_2_. The appearance of new and earlier eluting peaks (3.47 min) in the chromatograms could be due to the formation of melatonin metabolite products with more hydrophobicity and lower polarity that N(1)-acetyl-N(2)-formyl-5-methoxykynuramine (AFMK) (3.57 min) that could be achieved by treating MPO/melatonin with higher concentrations of H_2_O_2_. A hydroxylated intermediate in addition to AFMK was reported when melatonin was treated with H_2_O_2_ or neutrophils [23]. Additionally, activation of MPO by the diffused intra-oocyte H_2_O_2_ was confirmed utilizing the taurine chloramine assay. Indeed, incubation of 80 oocytes for 30 minutes with MPO in the presence of taurine caused the formation of a deeper blue color compared to control.

## Discussion

Here, we utilized a custom made H_2_O_2_-selective probe (L-shaped 5–15 μm tip) to precisely measure and quantitate the intra-oocyte cellular levels of H_2_O_2_, a signaling molecule that affects biological and physiological function of the oocyte. Our findings suggest that the H_2_O_2_ concentration of oocytes obtained from young animals (super-ovulated 8–14 week-old mice) is relatively high (1.0 + 0.07 μM), and that a significant portion appears to diffuse outside the oocyte. While the H_2_O_2_ levels remain reasonably constant during measurements, the intra-oocyte H_2_O_2_ concentration was reduced significantly (40–50%) when oocytes were pre-incubated with catalytic amounts of catalase, suggesting that these measurements were truly H_2_O_2_ centered rather than caused by an unknown interfering substance in our system. H_2_O_2_ diffusion out of the oocytes is also demonstrated through its ability to trigger the chlorinating activity of MPO in which HOCl alters metaphase-II mouse oocyte quality parameters of MT and CH. These alterations can be prevented by pre-incubation of oocytes with melatonin, a potent MPO inhibitor and HOCl scavenger [22]. These findings, in part, contribute to our establishment of the hypothesis that elevated levels of reactive oxygen species (ROS) such as H_2_O_2_ and HOCl as well as the MPO system directly or indirectly play a significant role in deteriorating oocyte quality.

In general, H_2_O_2_ is known to play a crucial role in both signaling and cellular regulation, and can be produced in all cellular compartments (mitochondria, cytosol, and peroxisome) from the enzymatic dismutation of O_2_^•−^ [39]. After reversible diffusion between the cells compartments, a substantial portion of the intra-cellular H_2_O_2_ (60–70%) is either converted enzymatically through glutathione peroxidase and/or catalase to water, consumed by non-enzymatic low molecular weight antioxidants such as reduced glutathione, or converted to other ROS in the presence of electron donors [8, 40]. Based on the model of the semipermeable membrane, which is applied to biological systems, molecular transport through membranes depends on the size, charge of molecules to be transported and membrane composition [41]. Hydrogen peroxide, unlike O_2_^•−^, is an uncharged and stable molecule, and therefore displays permeability in biological membranes. Hydrogen peroxide is thought to pass through biological membranes in a fashion similar to water, via limited diffusion as well as through specialized transport proteins called aquaporins [42]. Aquaporins similarly aid in the transportation of numerous other small uncharged and partially charged molecules including glycerol, urea, CO_2_, polyols, purines, pyrimidines, and nitrate [43, 44]. The rate of movement through these proteins is regulated and variable depending on a variety of factors including gradients [45]. Newer evidence also suggests certain aquaporins may be specific to certain molecules; H_2_O_2_-specific aquaporins have been described in yeast cells [46]. Based on theoretical modeling work it appears that under physiologic conditions, approximately 30–40% of intracellular H_2_O_2_ diffuses out of the cell [9, 46].

Quantitative measurement of H_2_O_2_ in rat liver cells was calculated using catalase levels to range from ~0.001 to ~0.1 μM in periods of low and high H_2_O_2_ generation respectively [47, 48]. In mitochondrial cells, mathematical modeling gave an estimate of 0.04 μM [49]. Similarly, steady-state levels of H_2_O_2_ measured from the extracellular environment of mammalian cell suspensions have ranged from 0.02 to 2 μM. Calculations based on the permeability and gradient of H_2_O_2_ estimated that intracellular concentrations would be approximately 0.002–0.2 μM [50, 51]. The use of an amperometric microsensor has also been described in the measurement of H_2_O_2_ levels in rat brain tissue, estimating the extra and intracellular concentration of H_2_O_2_ to be 2.0–4.0 μM and 0.2–0.4 μM respectively [52]. In summation, these results suggest an intracellular mammalian physiologic range of H_2_O_2_ to be 0.001–0.7 μM [53]. These levels have also been studied in their relationship to adverse cellular effects. Hydrogen peroxide levels above the 0.5–0.7 μM range were found to be associated with apoptosis in Jurkat T cells [54]. Earlier studies by different groups utilizing different methods have shown that the intracellular H_2_O_2_ concentration ranging from ~0.001 μM to a higher of ~0.1 μM during peak H_2_O_2_ generation [47, 48]. Our measured intra-oocyte H_2_O_2_ concentration was estimated to be ~1.0 μM and reduced to the half when the oocyte was pre-incubated with catalase. Therefore, our findings are consistent with results obtained from other cell types. However, these results are in contrast to findings by Tripathi et al. in which the mouse intra-oocyte levels of H_2_O_2_ were estimated to be approximately 80 ng/oocyte. Based on an oocyte volume of 249 pL [55], this H_2_O_2_ concentration at 9.45 M is an improbably high value and is not consistent with life [56]. Low concentration of exogenously added H_2_O_2_ exposed to the oocyte for short duration has no or little effect on oocyte quality whereas higher concentration and longer exposure to H_2_O_2_ deteriorates oocyte quality [1, 5]. Cumulus cells show some protection against lower H_2_O_2_ concentration, but this protection is lost at higher concentrations (Shaeib *et al*. unpublished results). As oocytes have the additional protective antioxidant machinery of the cumulus cell, a feature, which is not seen in other cell types, the oocyte may be able to accommodate or withstand higher concentrations of H_2_O_2_ than other cell types.

We believe that sufficient amounts of H_2_O_2_ are released from the oocyte to the extracellular milieu and triggered the chlorinating activity of MPO. The amount of HOCl generated from MPO under these conditions is known to be cytotoxic as indicated by its capability to deteriorate oocyte quality [26]. Melatonin has been shown to significantly protect oocyte quality against HOCl assault either through the inhibition of MPO or through the direct scavenging of HOCl [26]. Previously, we have shown the melatonin can inhibit the catalytic activity of MPO through its ability to compete with Cl^-^ and switch the MPO catalytic activity from a 2e^-^ oxidation of Cl^-^ to a 1e^-^ oxidation pathway [22]. In these conditions the enzyme maintains its peroxidase activity but it loses its chlorinating activity and the net result is melatonin oxidation [22]. Thus, the protection is limited by melatonin concentration and the rate of its consumption by MPO. Consistent with these findings, we recently have shown that exogenously added HOCl deteriorates oocyte quality in a manner comparable to our current observed results independent of the presence of cumulus cells [26]. Elevated levels of HOCl have many damaging effects including, but not limited to, the loss of mitochondrial DNA, the loss of the functional electron transport chains, as well as protein oxidation, and cause oxidative stress via hemoproteins heme destruction, protein aggregation, lipid peroxidation, change the membrane lipid composition, and lysis of the cell membrane leading to oocyte death [57]. Hypochlorous acid could also mediate the dysregulation of the overall antioxidant defense machinery, which could impact optimal chromatin decondensation at fertilization and, therefore, vary gene expression [23, 58–60]. Importantly, we have found that addition of melatonin protects against exogenous HOCl mediated damage [10].

Our evidence suggests that the deterioration of oocyte quality is mediated by MPO chlorinating activity rather than direct H_2_O_2_ insult. Hydrogen peroxide is a less powerful oxidant compared to HOCl. In physiologic circumstances, the exact nature of the relationship between H_2_O_2_ released from the oocyte and its interaction with cumulus cells is still under investigation. However, recently, we have shown that in the absence of cumulus cells, H_2_O_2_, through a mechanism that involves alteration in the MT and CH, deteriorates oocyte quality in a concentration dependent manner [5]. Cumulus cells provide protection against H_2_O_2_ insult at lower concentrations (>50 μM), but fail to protect the oocyte against higher concentrations (Shaeib *et al*. unpublished results). Therefore, we can speculate that disturbance in antioxidant machinery either by decreasing antioxidant enzymatic activity or exhaustion of small antioxidant molecules increases intra-oocyte H_2_O_2_ concentration and its diffusion to oocytes’ surrounding. These phenomena may explain failures in the fertilization process in some oocyte during intracytoplasmic sperm injection (ICSI), which requires in part good oocyte quality. Uncontrolled generation of intracellular H_2_O_2_ may lead to atresia, poorly formed zona pellucida, or abnormal eggs that have limited or no potential of further development [5, 61]. Our findings also link inflammatory conditions related to poor reproductive outcomes such as diabetes, endometriosis and others with oocyte aging or deterioration of oocyte quality from elevated MPO levels [62, 63]. For example, it has been shown that in advanced stage endometriosis compared with early stage, neutrophil activity with expression of MPO, and thus HOCl are higher secondary to either suppression of phagocytic activity or establishment of neovascularization [64]. Efforts have therefore been made to prevent deterioration in oocyte quality by supplementing the culture media with antioxidants, such as melatonin, caffeine, vitamin C and reduced glutathione (GSH) [65].

In conclusion, this investigation presents an oocyte-exclusive method for quantitation of H_2_O_2_ which is aimed at reducing interfering effects and providing the highest sensitivity and precision in H_2_O_2_ detection in a single oocyte. Finally, we link MPO with poor oocyte quality and poor reproductive outcomes in the setting of inflammatory conditions. Additionally, with growing evidence that melatonin can protect oocytes against deterioration; melatonin may be a potential target for therapeutic intervention.

## References

[1] Goud AP, Goud PT, Diamond MP, Gonik B, Abu-Soud HM. Reactive oxygen species and oocyte aging: role of superoxide, hydrogen peroxide, and hypochlorous acid. Free radical biology & medicine. 2008;44(7):1295–304. 18177745 10.1016/j.freeradbiomed.2007.11.014 PMC3416041

[2] Agarwal A, Aponte-Mellado A, Premkumar BJ, Shaman A, Gupta S. The effects of oxidative stress on female reproduction: a review. Reproductive biology and endocrinology: RB&E. 2012;10:49 10.1186/1477-7827-10-49 22748101 PMC3527168

[3] Gutteridge JM, Halliwell B. Comments on review of Free Radicals in Biology and Medicine, second edition, by Barry Halliwell and John M. C. Gutteridge. Free radical biology & medicine. 1992;12(1):93–5.1537574

[4] Schallreuter KU, Moore J, Wood JM, Beazley WD, Gaze DC, Tobin DJ, et al In vivo and in vitro evidence for hydrogen peroxide (H2O2) accumulation in the epidermis of patients with vitiligo and its successful removal by a UVB-activated pseudocatalase. The journal of investigative dermatology Symposium proceedings / the Society for Investigative Dermatology, Inc [and] European Society for Dermatological Research. 1999;4(1):91–6. 10.1038/sj.jidsp.5640189 10537016

[5] Shaeib F, Banerjee J, Maitra D, Diamond MP, Abu-Soud HM. Impact of hydrogen peroxide-driven Fenton reaction on mouse oocyte quality. Free radical biology & medicine. 2013;58:154–9. 23261938 10.1016/j.freeradbiomed.2012.12.007 PMC4482232

[6] Choi WJ, Banerjee J, Falcone T, Bena J, Agarwal A, Sharma RK. Oxidative stress and tumor necrosis factor-alpha-induced alterations in metaphase II mouse oocyte spindle structure. Fertility and sterility. 2007;88(4 Suppl):1220–31. 17601599 10.1016/j.fertnstert.2007.02.067

[7] Bienert GP, Schjoerring JK, Jahn TP. Membrane transport of hydrogen peroxide. Biochimica et biophysica acta. 2006;1758(8):994–1003. 16566894 10.1016/j.bbamem.2006.02.015

[8] Antunes F, Cadenas E. Estimation of H2O2 gradients across biomembranes. FEBS letters. 2000;475(2):121–6. 10858501 10.1016/s0014-5793(00)01638-0

[9] Makino N, Sasaki K, Hashida K, Sakakura Y. A metabolic model describing the H2O2 elimination by mammalian cells including H2O2 permeation through cytoplasmic and peroxisomal membranes: comparison with experimental data. Biochimica et biophysica acta. 2004;1673(3):149–59. 15279886 10.1016/j.bbagen.2004.04.011

[10] Maitra D, Abdulhamid I, Diamond MP, Saed GM, Abu-Soud HM. Melatonin attenuates hypochlorous acid-mediated heme destruction, free iron release, and protein aggregation in hemoglobin. Journal of pineal research. 2012;53(2):198–205. 10.1111/j.1600-079X.2012.00988.x 22462755

[11] Klebanoff SJ. Myeloperoxidase: friend and foe. Journal of leukocyte biology. 2005;77(5):598–625. 15689384 10.1189/jlb.1204697

[12] Hampton MB, Kettle AJ, Winterbourn CC. Inside the neutrophil phagosome: oxidants, myeloperoxidase, and bacterial killing. Blood. 1998;92(9):3007–17. 9787133

[13] Cieutat AM, Lobel P, August JT, Kjeldsen L, Sengelov H, Borregaard N, et al Azurophilic granules of human neutrophilic leukocytes are deficient in lysosome-associated membrane proteins but retain the mannose 6-phosphate recognition marker. Blood. 1998;91(3):1044–58. 9446668

[14] Davies MJ, Hawkins CL, Pattison DI, Rees MD. Mammalian heme peroxidases: from molecular mechanisms to health implications. Antioxidants & redox signaling. 2008;10(7):1199–234. 18331199 10.1089/ars.2007.1927

[15] Blair SA, Kyaw-Tun T, Young IS, Phelan NA, Gibney J, McEneny J. Oxidative stress and inflammation in lean and obese subjects with polycystic ovary syndrome. The Journal of reproductive medicine. 2013;58(3–4):107–14. 23539878

[16] da Silva CM, Vilaca Belo A, Passos Andrade S, Peixoto Campos P, Cristina Franca Ferreira M, Lopes da Silva-Filho A, et al Identification of local angiogenic and inflammatory markers in the menstrual blood of women with endometriosis. Biomedicine & pharmacotherapy = Biomedecine & pharmacotherapie. 2014. 10.1016/j.biopha.2014.08.005 25218120

[17] Binder V, Ljubojevic S, Haybaeck J, Holzer M, El-Gamal D, Schicho R, et al The myeloperoxidase product hypochlorous acid generates irreversible high-density lipoprotein receptor inhibitors. Arteriosclerosis, thrombosis, and vascular biology. 2013;33(5):1020–7. 10.1161/ATVBAHA.113.301235 23493288 PMC4163628

[18] Nicholls SJ, Hazen SL. Myeloperoxidase, modified lipoproteins, and atherogenesis. Journal of lipid research. 2009;50 Suppl:S346–51. 10.1194/jlr.R800086-JLR200 19091698 PMC2674690

[19] Fletcher NM, Jiang Z, Ali-Fehmi R, Levin NK, Belotte J, Tainsky MA, et al Myeloperoxidase and free iron levels: potential biomarkers for early detection and prognosis of ovarian cancer. Cancer biomarkers: section A of Disease markers. 2011;10(6):267–75. 10.3233/CBM-2012-0255 22820082 PMC13016252

[20] Navarro-Alarcon M, Ruiz-Ojeda FJ, Blanca-Herrera RM, MM AS, Acuna-Castroviejo D, Fernandez-Vazquez G, et al Melatonin and metabolic regulation: a review. Food & function. 2014. 10.1039/c4fo00317a 25207999

[21] Reiter RJ, Tan DX, Galano A. Melatonin: Exceeding Expectations. Physiology. 2014;29(5):325–33. 10.1152/physiol.00011.2014 25180262

[22] Galijasevic S, Abdulhamid I, Abu-Soud HM. Melatonin is a potent inhibitor for myeloperoxidase. Biochemistry. 2008;47(8):2668–77. 10.1021/bi702016q 18237195

[23] Ximenes VF, Silva SO, Rodrigues MR, Catalani LH, Maghzal GJ, Kettle AJ, et al Superoxide-dependent oxidation of melatonin by myeloperoxidase. The Journal of biological chemistry. 2005;280(46):38160–9. 16148002 10.1074/jbc.M506384200

[24] Malle E, Furtmuller PG, Sattler W, Obinger C. Myeloperoxidase: a target for new drug development? British journal of pharmacology. 2007;152(6):838–54. 17592500 10.1038/sj.bjp.0707358 PMC2078229

[25] Maitra D, Shaeib F, Abdulhamid I, Abdulridha RM, Saed GM, Diamond MP, et al Myeloperoxidase acts as a source of free iron during steady-state catalysis by a feedback inhibitory pathway. Free radical biology & medicine. 2013;63:90–8. 23624305 10.1016/j.freeradbiomed.2013.04.009 PMC3863623

[26] Banerjee J, Maitra D, Diamond MP, Abu-Soud HM. Melatonin prevents hypochlorous acid-induced alterations in microtubule and chromosomal structure in metaphase-II mouse oocytes. Journal of pineal research. 2012;53(2):122–8. 10.1111/j.1600-079X.2012.00977.x 22304486

[27] Banerjee J, Shaeib F, Maitra D, Saed GM, Dai J, Diamond MP, et al Peroxynitrite affects the cumulus cell defense of metaphase II mouse oocytes leading to disruption of the spindle structure in vitro. Fertility and sterility. 2013;100(2):578–84 e1 10.1016/j.fertnstert.2013.04.030 23721714

[28] Proteasa G, Tahboub YR, Galijasevic S, Raushel FM, Abu-Soud HM. Kinetic evidence supports the existence of two halide binding sites that have a distinct impact on the heme iron microenvironment in myeloperoxidase. Biochemistry. 2007;46(2):398–405. 17209550 10.1021/bi0609725

[29] Rakita RM, Michel BR, Rosen H. Differential inactivation of Escherichia coli membrane dehydrogenases by a myeloperoxidase-mediated antimicrobial system. Biochemistry. 1990;29(4):1075–80. 1692736 10.1021/bi00456a033

[30] Tahboub YR, Galijasevic S, Diamond MP, Abu-Soud HM. Thiocyanate modulates the catalytic activity of mammalian peroxidases. The Journal of biological chemistry. 2005;280(28):26129–36. 15894800 10.1074/jbc.M503027200

[31] Andrews PC, Krinsky NI. A kinetic analysis of the interaction of human myeloperoxidase with hydrogen peroxide, chloride ions, and protons. The Journal of biological chemistry. 1982;257(22):13240–5. 6292181

[32] Goud PT, Goud AP, Najafi T, Gonik B, Diamond MP, Saed GM, et al Direct real-time measurement of intra-oocyte nitric oxide concentration in vivo. PloS one. 2014;9(6):e98720 10.1371/journal.pone.0098720 24887331 PMC4041775

[33] Sliskovic I, Abdulhamid I, Sharma M, Abu-Soud HM. Analysis of the mechanism by which tryptophan analogs inhibit human myeloperoxidase. Free radical biology & medicine. 2009;47(7):1005–13. 19596067 10.1016/j.freeradbiomed.2009.07.007

[34] Jimenez-Jimenez FJ, Orti-Pareja M, Molina-Arjona JA. [Mitochondrial changes in neurodegenerative diseases]. Rev Neurol. 1998;26 Suppl 1:S112–7. 9810599

[35] Abu-Soud HM, Maitra D, Shaeib F, Khan SN, Byun J, Abdulhamid I, et al Disruption of heme-peptide covalent cross-linking in mammalian peroxidases by hypochlorous acid. J Inorg Biochem. 2014;140:245–54. 10.1016/j.jinorgbio.2014.06.018 25193127 PMC4449957

[36] Goud PT, Goud AP, Diamond MP, Gonik B, Abu-Soud HM. Nitric oxide extends the oocyte temporal window for optimal fertilization. Free radical biology & medicine. 2008;45(4):453–9. 18489913 10.1016/j.freeradbiomed.2008.04.035 PMC3786211

[37] Goud P, Rybouchkin A, De Sutter P, Dhont M. Fine points of technique—ICSI. Fertility and sterility. 1997;67(5):979–80. 9130917 10.1016/s0015-0282(97)90147-3

[38] Goud AP, Goud PT, Diamond MP, Van Oostveldt P, Hughes MR. Microtubule turnover in ooplasm biopsy reflects ageing phenomena in the parent oocyte. Reprod Biomed Online. 2005;11(1):43–52. 16102286 10.1016/s1472-6483(10)61297-7

[39] Pick E, Keisari Y. Superoxide anion and hydrogen peroxide production by chemically elicited peritoneal macrophages—induction by multiple nonphagocytic stimuli. Cellular immunology. 1981;59(2):301–18. 6269759 10.1016/0008-8749(81)90411-1

[40] Presnell CE, Bhatti G, Numan LS, Lerche M, Alkhateeb SK, Ghalib M, et al Computational insights into the role of glutathione in oxidative stress. Current neurovascular research. 2013;10(2):185–94. 23469953 10.2174/1567202611310020011

[41] Li L, Shi X, Guo X, Li H, Xu C. Ionic protein-lipid interaction at the plasma membrane: what can the charge do? Trends in biochemical sciences. 2014;39(3):130–40. 10.1016/j.tibs.2014.01.002 24534649

[42] Vieceli Dalla Sega F, Zambonin L, Fiorentini D, Rizzo B, Caliceti C, Landi L, et al Specific aquaporins facilitate Nox-produced hydrogen peroxide transport through plasma membrane in leukaemia cells. Biochimica et biophysica acta. 2014;1843(4):806–14. 10.1016/j.bbamcr.2014.01.011 24440277

[43] Rambow J, Wu B, Ronfeldt D, Beitz E. Aquaporins with anion/monocarboxylate permeability: mechanisms, relevance for pathogen-host interactions. Frontiers in pharmacology. 2014;5:199 10.3389/fphar.2014.00199 25225485 PMC4150397

[44] Tsukaguchi H, Shayakul C, Berger UV, Mackenzie B, Devidas S, Guggino WB, et al Molecular characterization of a broad selectivity neutral solute channel. The Journal of biological chemistry. 1998;273(38):24737–43. 9733774 10.1074/jbc.273.38.24737

[45] Conde A, Diallinas G, Chaumont F, Chaves M, Geros H. Transporters, channels, or simple diffusion? Dogmas, atypical roles and complexity in transport systems. The international journal of biochemistry & cell biology. 2010;42(6):857–68. 20026419 10.1016/j.biocel.2009.12.012

[46] Henzler T, Steudle E. Transport and metabolic degradation of hydrogen peroxide in Chara corallina: model calculations and measurements with the pressure probe suggest transport of H(2)O(2) across water channels. Journal of experimental botany. 2000;51(353):2053–66. 11141179 10.1093/jexbot/51.353.2053

[47] Chance B, Sies H, Boveris A. Hydroperoxide metabolism in mammalian organs. Physiological reviews. 1979;59(3):527–605. 37532 10.1152/physrev.1979.59.3.527

[48] Oshino N, Chance B, Sies H, Bucher T. The role of H 2 O 2 generation in perfused rat liver and the reaction of catalase compound I and hydrogen donors. Archives of biochemistry and biophysics. 1973;154(1):117–31. 4347674 10.1016/0003-9861(73)90040-4

[49] Antunes F, Salvador A, Marinho HS, Alves R, Pinto RE. Lipid peroxidation in mitochondrial inner membranes. I. An integrative kinetic model. Free radical biology & medicine. 1996;21(7):917–43. 8937879 10.1016/s0891-5849(96)00185-2

[50] Boveris A, Alvarez S, Bustamante J, Valdez L. Measurement of superoxide radical and hydrogen peroxide production in isolated cells and subcellular organelles. Methods in enzymology. 2002;349:280–7. 11912917 10.1016/s0076-6879(02)49342-1

[51] Mueller S. Sensitive and nonenzymatic measurement of hydrogen peroxide in biological systems. Free radical biology & medicine. 2000;29(5):410–5. 11020662 10.1016/s0891-5849(00)00261-6

[52] Kulagina NV, Michael AC. Monitoring hydrogen peroxide in the extracellular space of the brain with amperometric microsensors. Analytical chemistry. 2003;75(18):4875–81. 14674466 10.1021/ac034573g

[53] Stone JR, Yang S. Hydrogen peroxide: a signaling messenger. Antioxidants & redox signaling. 2006;8(3–4):243–70. 16677071 10.1089/ars.2006.8.243

[54] Antunes F, Cadenas E. Cellular titration of apoptosis with steady state concentrations of H(2)O(2): submicromolar levels of H(2)O(2) induce apoptosis through Fenton chemistry independent of the cellular thiol state. Free radical biology & medicine. 2001;30(9):1008–18. 11316581 10.1016/s0891-5849(01)00493-2

[55] Pogorelov AG, Pogorelova VN. Quantitative tomography of early mouse embryos: laser scanning microscopy and 3D reconstruction. Journal of microscopy. 2008;232(1):36–43. 10.1111/j.1365-2818.2008.02077.x 19017199

[56] Tripathi A, Khatun S, Pandey AN, Mishra SK, Chaube R, Shrivastav TG, et al Intracellular levels of hydrogen peroxide and nitric oxide in oocytes at various stages of meiotic cell cycle and apoptosis. Free radical research. 2009;43(3):287–94. 10.1080/10715760802695985 19184696

[57] Maitra D, Byun J, Andreana PR, Abdulhamid I, Diamond MP, Saed GM, et al Reaction of hemoglobin with HOCl: mechanism of heme destruction and free iron release. Free radical biology & medicine. 2011;51(2):374–86. 21549834 10.1016/j.freeradbiomed.2011.04.011 PMC3863628

[58] Souza CE, Maitra D, Saed GM, Diamond MP, Moura AA, Pennathur S, et al Hypochlorous acid-induced heme degradation from lactoperoxidase as a novel mechanism of free iron release and tissue injury in inflammatory diseases. PloS one. 2011;6(11):e27641 10.1371/journal.pone.0027641 22132121 PMC3222650

[59] de Matos DG, Gasparrini B, Pasqualini SR, Thompson JG. Effect of glutathione synthesis stimulation during in vitro maturation of ovine oocytes on embryo development and intracellular peroxide content. Theriogenology. 2002;57(5):1443–51. 12054203 10.1016/s0093-691x(02)00643-x

[60] Cheng WM, An L, Wu ZH, Zhu YB, Liu JH, Gao HM, et al Effects of disulfide bond reducing agents on sperm chromatin structural integrity and developmental competence of in vitro matured oocytes after intracytoplasmic sperm injection in pigs. Reproduction. 2009;137(4):633–43. 10.1530/REP-08-0143 19155332

[61] Hennet ML, Yu HY, Combelles CM. Follicular fluid hydrogen peroxide and lipid hydroperoxide in bovine antral follicles of various size, atresia, and dominance status. Journal of assisted reproduction and genetics. 2013;30(3):333–40. 10.1007/s10815-012-9925-5 23315290 PMC3607686

[62] Saito H, Seino T, Kaneko T, Nakahara K, Toya M, Kurachi H. Endometriosis and oocyte quality. Gynecologic and obstetric investigation. 2002;53 Suppl 1:46–51. 11834868 10.1159/000049424

[63] Mills JL, Knopp RH, Simpson JL, Jovanovic-Peterson L, Metzger BE, Holmes LB, et al Lack of relation of increased malformation rates in infants of diabetic mothers to glycemic control during organogenesis. The New England journal of medicine. 1988;318(11):671–6. 3344018 10.1056/NEJM198803173181104

[64] Riley CF, Moen MH, Videm V. Inflammatory markers in endometriosis: reduced peritoneal neutrophil response in minimal endometriosis. Acta obstetricia et gynecologica Scandinavica. 2007;86(7):877–81. 17611835 10.1080/00016340701417398

[65] Singh AK, Chattopadhyay R, Chakravarty B, Chaudhury K. Markers of oxidative stress in follicular fluid of women with endometriosis and tubal infertility undergoing IVF. Reproductive toxicology. 2013;42:116–24. 10.1016/j.reprotox.2013.08.005 23994512

